# Supplementary material for: Identifying contamination with advanced visualization and analysis practices: metagenomic approaches for eukaryotic genome assemblies
Source: PeerJ. 2016 Mar 29;4:e1839. doi: 10.7717/peerj.1839 (PMC4824900; doi:10.7717/peerj.1839)
Supplement: Supplemental Information 1 [file peerj-04-1839-s001.pdf]

SUPPLEMENTARY INFORMATION

Tom O. Delmont[1] and A. Murat Eren[1,2*]

*Correspondence:
meren@uchicago.edu

[1]The Department of Medicine, The University of Chicago, Chicago, 60637 IL, USA

[2]Josephine Bay Paul Center for Comparative Molecular Biology and Evolution, Marine Biological Laboratory, Woods Hole, 02543 MA, USA

Estimating the number of bacterial genomes occurring in an assembly using single-copy genes and the 'mode'

Accurate reconstruction of bacterial genomes that occur in an assembly depends on many factors, including the complexity of the metagenome, the number of samples that can be exploited for differential coverage patterns for contigs, or the approach that will generate genome bins. However, even before processing the assembly results for binning, it is possible to acquire an estimation for the approximate number of bacterial genomes an assembly contains.

A set of genes that are found in most bacterial genomes as a single-copy are commonly used to predict the completeness of bacterial genome bins identified from metagenomic data [1, 2, 3, 4]. By relying on the same assumption, we use single-copy genes found in an assembly as a proxy to the total number of complete bacterial genomes the assembly contains. Our approach relies on the 'mode' (i.e., the most frequently occurring number) of the array of the number of single-copy gene hits in an assembly. Three examples to elucidate the relevance of this simple statistical metric to predict the number of genomes in an assembly follows. For each example, we used three single-copy gene collections published by Alneberg et al. [5] with 34 genes, Creevey et al. [6] with 40 genes, and Campbell et al. [2] with 139 genes.

Gut metagenome

Sharon et al. [7] generated 11 shotgun metagenomes from daily sampling of an infant's gut. Figure 1 shows the occurrence of single-copy genes in the raw co-assembly results of these data. The mode of each array of single-copy gene hits

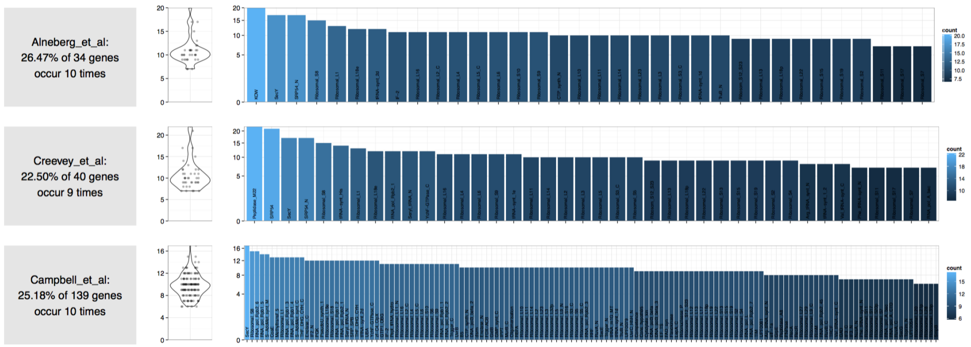

Figure 1 Occurrence of single copy genes in Sharon et al. (2013) co-assembly. Each bar represents the squared-root normalized number of significant hits per single-copy gene in each collection. The same information is visualized as box-plots on the left side of each plot.

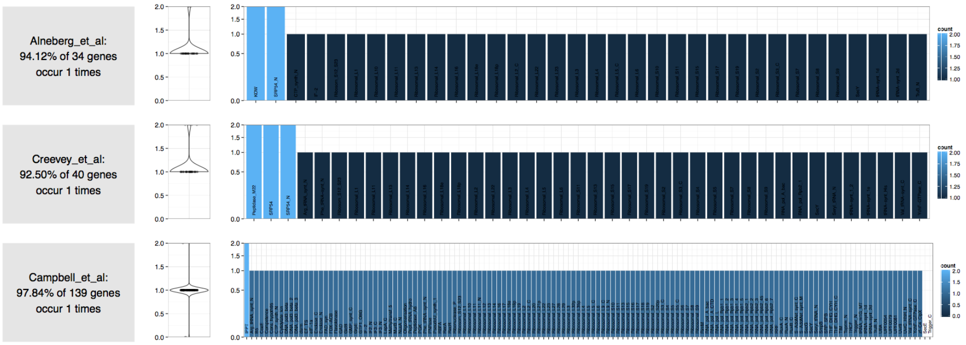

Figure 2 Occurrence of single-copy genes in an assembly of a cultivar genome

predict 10, 9, and 10 bacterial genomes, respectively. A detailed analysis of this dataset by Sharon et al. [7], and later our group [4] identified 12 bacterial draft genomes, 8 of which were complete or near complete.

Cultivar genome

As a control, Figure 2 shows the analysis of an assembly that is generated from a bacterial cultivar (unpublished). The mode in this case correctly predicts the occurrence of a single genome in the raw assembly results.

Ocean metagenome

In contrast, Figure 3 displays the distribution of bacterial single-copy genes in the raw assembly results of an ocean sample (unpublished). Unlike the infant gut and cultivar assemblies, this dataset represents a much more diverse sample. Yet, predictions from the three single-copy gene collections show remarkable stability despite the increased complexity: the mode of single-copy gene hits for each collection predicts 451, 451, and 431 genomes in this assembly, respectively.

Our results suggest that this simple metric offers a reliable first approximation to the expected number of bacterial genomes in an assembly. This information can be helpful to researchers who wish to gain a quick insight regarding the extent of bacterial contamination in their eukaryotic genome assembly results. Similarly, this information can also be useful for microbiologists as it provides a quick means to understand the complexity of a given metagenomic assembly. Finally, it can be used to make educated guesses for the expected number of genomes automated binning tools should report from a given metagenomic assembly, which can be beneficial for benchmarking and quality assurance purposes.

[1]The Department of Medicine, The University of Chicago, Chicago, 60637 IL, USA. [2]Josephine Bay Paul Center for Comparative Molecular Biology and Evolution, Marine Biological Laboratory, Woods Hole, 02543 MA, USA.

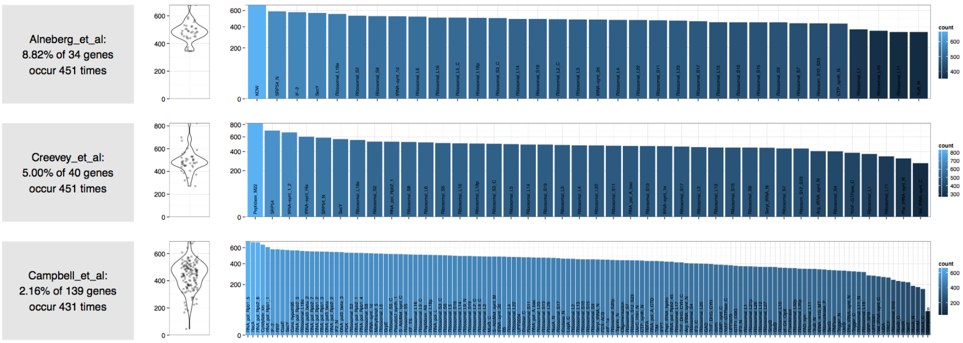

Figure 3 Occurrence of single-copy genes in an assembly of an ocean sample

References

1. Wu, M., Eisen, J.A.: A simple, fast, and accurate method of phylogenomic inference. Genome Biology **9**(10), 151 (2008). doi:10.1186/gb-2008-9-10-r151

2. Campbell, J.H., O'Donoghue, P., Campbell, A.G., Schwientek, P., Sczyrba, A., Woyke, T., Söll, D., Podar, M.: UGA is an additional glycine codon in uncultured SR1 bacteria from the human microbiota. Proceedings of the National Academy of Sciences of the United States of America **110**(14), 5540–5 (2013). doi:10.1073/pnas.1303090110

3. Parks, D.H., Imelfort, M., Skennerton, C.T., Hugenholtz, P., Tyson, G.W.: CheckM: assessing the quality of microbial genomes recovered from isolates, single cells, and metagenomes. Genome research **25**(7), 1043–55 (2015). doi:10.1101/gr.186072.114

4. Eren, A.M., Esen, Ö.C., Quince, C., Vineis, J.H., Morrison, H.G., Sogin, M.L., Delmont, T.O.: Anvi'o: an advanced analysis and visualization platform for 'omics data. PeerJ **3**, 1319 (2015). doi:10.7717/peerj.1319

5. Alneberg, J., Bjarnason, B.S., de Bruijn, I., Schirmer, M., Quick, J., Ijaz, U.Z., Lahti, L., Loman, N.J., Andersson, A.F., Quince, C.: Binning metagenomic contigs by coverage and composition. Nature Methods **11**(11), 1144–1146 (2014). doi:10.1038/nmeth.3103

6. Creevey, C.J., Doerks, T., Fitzpatrick, D.A., Raes, J., Bork, P.: Universally distributed single-copy genes indicate a constant rate of horizontal transfer. PloS one **6**(8), 22099 (2011). doi:10.1371/journal.pone.0022099

7. Sharon, I., Morowitz, M.J., Thomas, B.C., Costello, E.K., Relman, D.A., Banfield, J.F.: Time series community genomics analysis reveals rapid shifts in bacterial species, strains, and phage during infant gut colonization. Genome research **23**(1), 111–20 (2013). doi:10.1101/gr.142315.112
