# Supplementary figures and images for: Identifying contamination with advanced visualization and analysis practices: metagenomic approaches for eukaryotic genome assemblies
Source: PeerJ. 2016 Mar 29;4:e1839. doi: 10.7717/peerj.1839 (PMC4824900; doi:10.7717/peerj.1839)

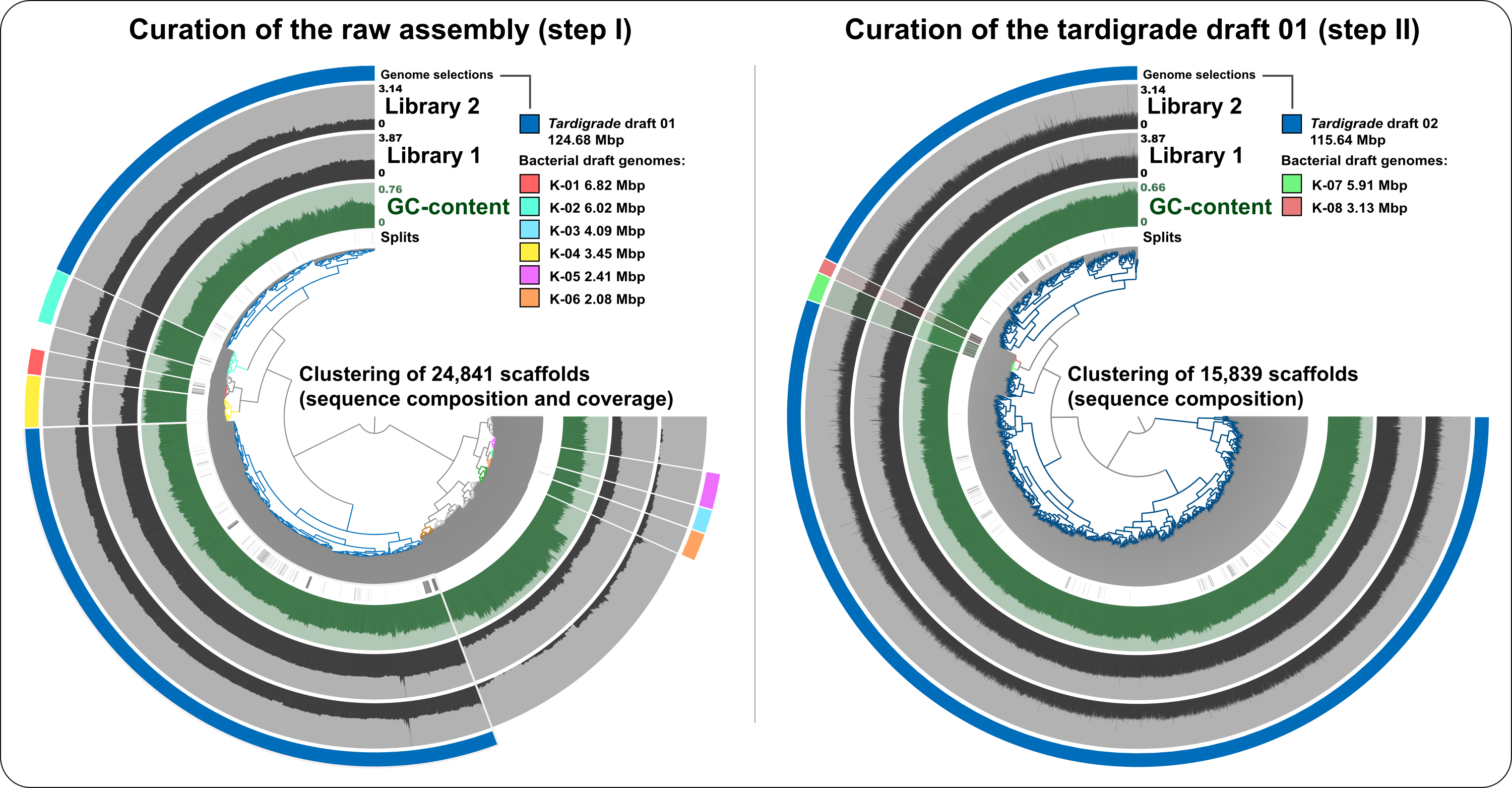

Supplement: Figure S1 — In the left panel (curation step I), 24,841 scaffolds that were longer than 1 kbp from the raw assembly were clustered based on sequence composition and coverage values in data from the two Illumina sequencing libraries (the inner dendrogram). Scaffolds longer than 40 kbp were split into sections of 20 kbp for visualization purposes. The second layer shows the GC-content for each scaffold. The next two view layers represent the log-normalized mean coverage values for scaffolds in the two sequencing datasets. Finally, our scaffold selections (tardigrade draft 01 and six bacterial draft genomes) are displayed in the outer layer. In the right panel (curation step II), the 15,839 scaffolds from the tardigrade selection from step I were clustered based on sequence composition only for more precise curation. Additional scaffold selections (tardigrade draft 02 and two bacterial draft genomes) are displayed in the outer layer. [file peerj-04-1839-s002.png]
